# Combined free nitrous acid and hydrogen peroxide pre-treatment of waste activated sludge enhances methane production via organic molecule breakdown

**DOI:** 10.1038/srep16631

**Published:** 2015-11-13

**Authors:** Tingting Zhang, Qilin Wang, Liu Ye, Damien Batstone, Zhiguo Yuan

**Affiliations:** 1 Advanced Water Management Centre (AWMC), The University of Queensland, QLD 4072, Australia; 2 School of Chemical Engineering, The University of Queensland, QLD 4072, Australia

## Abstract

This study presents a novel pre-treatment strategy using combined free nitrous acid (FNA i.e. HNO_2_) and hydrogen peroxide (H_2_O_2_) to enhance methane production from WAS, with the mechanisms investigated bio-molecularly. WAS from a full-scale plant was treated with FNA alone (1.54 mg N/L), H_2_O_2_ alone (10–80 mg/g TS), and their combinations followed by biochemical methane potential tests. Combined FNA and H_2_O_2_ pre-treatment substantially enhanced methane potential of WAS by 59–83%, compared to 13–23% and 56% with H_2_O_2_ pre-treatment alone and FNA pre-treatment alone respectively. Model-based analysis indicated the increased methane potential was mainly associated with up to 163% increase in rapidly biodegradable fraction with combined pre-treatment. The molecular weight distribution and chemical structure analyses revealed the breakdown of soluble macromolecules with the combined pre-treatment caused by the deamination and oxidation of the typical functional groups in proteins, polysaccharides and phosphodiesters. These changes likely improved the biodegradability of WAS.

Anaerobic digestion (AD) has been widely used in waste activated sludge (WAS) treatment due to its ability to generate methane while reducing sludge volume^1^,^2^. However, the methane production is often limited by the poor biodegradability of WAS^1^. Therefore, many a pre-treatment strategies including mechanical, heat and chemical pre-treatment have been developed to improve methane production through cells lysis and/or extracellular polymeric substances (EPS) matrix disruption with the release of intracellular and extracellular constituents which can be biodegraded readily via digestion, thereby promoting methane production^3^,^4^,^5^,^6^,^7^,^8^. However, it has been noted that most of the aforementioned approaches are cost intensive due to high energy and/or chemical requirements^6^.

Free nitrous acid (FNA i.e. HNO_2_), a renewable and low cost chemical that can be produced on site by nitritation of the anaerobic digestion liquor^9^ has been demonstrated to be a strong biocidal agent, and be effective in enhancing WAS biodegradability and methane production^10^,^11^,^12^. The methane production from a full-scale WAS was improved up to 30% at a digestion time of 20 days with FNA pre-treatment at 2.1 mg N/L for 24 hours compared to that without FNA pre-treatment both economically and environmentally friendly^11^.

Recently, the *in situ* production of H_2_O_2_ from wastewater through a bio-electrochemical system has been proposed and tested^13^. Previous studies have demonstrated that combined FNA and H_2_O_2_ pre-treatment could further destroy microbial species and anaerobic wastewater biofilms compared with FNA pre-treatment alone^14^,^15^. This has been attributed to the generation of peroxynitrite (ONOO^−^) and highly reactive radicals such as NO_2_^•^ and HO^•^ from the reaction between FNA and H_2_O_2_^16^. Peroxynitrite and its radicals are powerful oxidants that are able to destroy cell components such as protein, DNA and membrane phospholipids, and also degrade EPS^17^,^18^,^19^. Therefore, these research outcomes led to the hypothesis that incorporating H_2_O_2_ into FNA-based sludge treatment strategy could potentially be more effective than FNA pre-treatment alone in enhancing methane production from WAS.

This study aims to: (1) evaluate the effect of combined FNA and H_2_O_2_ pre-treatment on methane production from WAS, and (2) identify the mechanisms responsible for the improved methane production. Full-scale WAS was subjected to i) no-chemical pre-treatment, ii) FNA pre-treatment alone, iii) H_2_O_2_ pre-treatment alone, and iv) Combined FNA and H_2_O_2_ pre-treatment. WAS solubilisation and biochemical methane potential (BMP) were assessed and compared. The molecular weight (MW) distribution measurement and chemical structure analysis of the soluble substances with and without chemical pre-treatment were then used in combination with the model-based analysis, to uncover the mechanisms that led to the improved performance.

## Results

### Effect of pre-treatment on biochemical methane production

**Figure 1:**
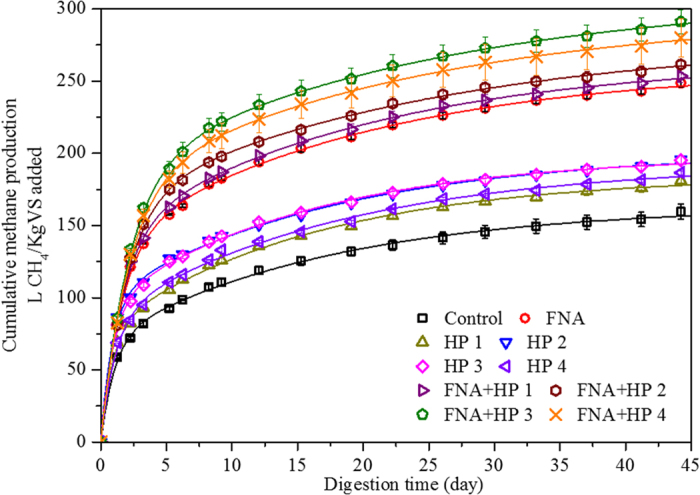
Measured and simulated methane production in the BMP tests (symbols represent experimental measurements; solid lines represent model fit using a two-substrate model. Error bars show standard errors).

The cumulative and modelled methane production from all samples over the 44-day BMP tests is shown in Fig. 1. All pre-treatment methods resulted in an increase in methane production throughout the tests. H_2_O_2_ pre-treatment alone resulted in a moderate increase in methane production, with relatively higher increases at H_2_O_2_ dosages of 30 to 50 mg/g TS than at lower (10 mg/g TS) and higher (80 mg/g TS) dosages. FNA pre-treatment alone achieved higher methane production than both control and H_2_O_2_ pre-treatment alone, which indicates that FNA pre-treatment alone is significantly more effective in enhancing methane production than H_2_O_2_ pre-treatment alone. The combined pre-treatment with FNA and H_2_O_2_, however, achieved the highest methane production of all pre-treatments used in this study.

The effectiveness of pre-treatments in enhancing methane production was reflected in the B_0_ results (Table 1). H_2_O_2_ pre-treatment alone enhanced methane potential by around 22% at 30 and 50 mg H_2_O_2_/g TS, with other dosage levels producing slightly less enhancement (13% and 17%, respectively, for dosages at 10 and 80 mg H_2_O_2_/g TS). FNA pre-treatment alone significantly increased methane potential by 56%, which is consistent with but higher than the result (30%) obtained in Wang, *et al.*^11^. It should be noted that the difference in WAS properties was likely responsible for the higher methane potential in this study. The combined FNA and H_2_O_2_ pre-treatment increased methane potential by 59–83%, achieving the highest level with FNA and H_2_O_2_ at 50 mg/g TS; higher or lower H_2_O_2_ dosages resulted in less enhancement of methane potential (B_0_).

### Biochemical methane potential (BMP) modelling results

Values of methane potentials (B_0,rapid_, B_0,slow_), hydrolysis rates (k_rapid_, k_slow_) and degradation extents (Y_rapid_, Y_slow_) were estimated, as summarized in Table 1. 95% confidence regions for the different parameter combinations were investigated to evaluate their identifiability as shown in Figs S1–S6 in Supplementary Information (SI). The linear confidence intervals (error bars) exceeded the non-linear regions because the former was the estimates through four-parameter estimation while the latter was estimated through two-parameter estimation by fixating the other two parameters at the estimates from four-parameter estimation. The lower degree of freedom and increased localized error function in four-parameter estimation may result in the over-estimation of the linear confidence intervals. However, the overall 95% confidence regions for all the six pairs are small, with mean values lying at the center and the 95% confidence intervals for all the individual parameters are generally within 10% of the estimated value. These indicate that the parameters are well identifiable and the estimated values are reliable.

k_slow_ did not show any significant variation with- or without chemical pre-treatment (0.04–0.06 d^−1^); whereas k_rapid_ varied substantially and systematically. Specifically, k_rapid_ averaged at 1.0 d^−1^ (±0.1) in the control and with H_2_O_2_ pre-treatment alone, and at 0.5 d^−1^ (±0.1) where FNA was used. One possibility is that the additional substances released in FNA pre-treatment are more slowly degradable than those released in the control and with H_2_O_2_ pre-treatment alone. The other possibility is that the additional release of rapidly degradable substances exceed the inoculum capacity to convert them into methane at the maximum rate, resulting in a reduced apparent rate as shown in Batstone, *et al.*^20^. Regardless of either of these issues, the B_0_ values remain representative of the rapid- and slowly degradable fractions.

**Figure 2:**
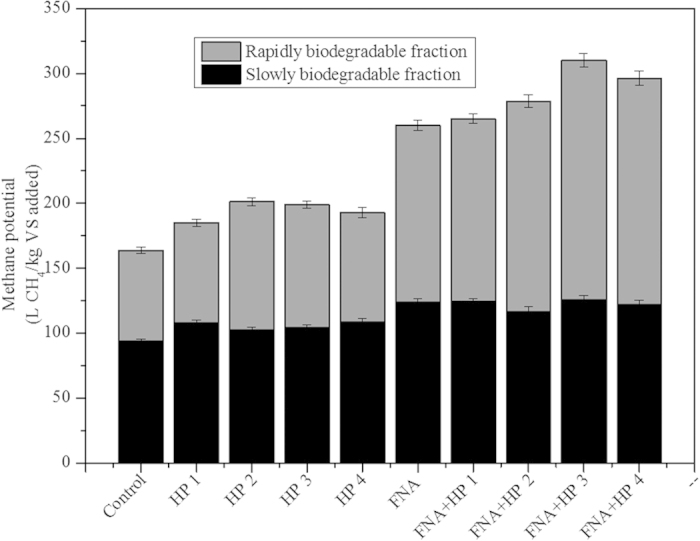
Modelled methane potential from different fractions of substrate.

B_0,total_ was enhanced substantially with all pre-treatments. In terms of B_0,rapid_, H_2_O_2_ pre-treatment alone led to an increase of 41% at H_2_O_2_ dosage of 30 mg/g TS compared with the case of control (from 70 L CH_4_/kg VS to 99 L CH_4_/kg VS). The other three dosages resulted in improved B_0,rapid_ by 10–21% (increased by 77–85 L CH_4_/kg VS). The B_0,rapid_ value in the case of FNA pre-treatment alone was 136 L CH_4_/kg VS, substantially higher than that in control (70 L CH_4_/kg VS). The B_0,rapid_ from the combined pre-treatment was further enhanced to 141–185 L CH_4_/kg VS, which corresponded to an improvement of 100–163% compared with that from the control. The highest improvement (163%) in B_0,rapid_ was achieved at the H_2_O_2_ dosage of 50 mg/g TS and FNA concentration of 1.54 mg N/L. B_0,slow_ with all pre-treatment was enhanced in comparison with that of control. The FNA pre-treatment and all the combined pre-treatments, regardless of the H_2_O_2_ level, resulted in very similar increases in B_0,slow_ (from 24% to 33%), and so did the H_2_O_2_ pre-treatment alone (from 6% to 13%). Y_rapid_ and Y_slow_ showed similar trends to B_0,rapid_ and B_0,slow_. These results collectively indicate that the improved performance is accredited to the increase of both rapidly and slowly biodegradable fractions but mainly from the rapidly biodegradable fraction, implying the conversion of non-biodegradable fraction into both rapidly and slowly biodegradable fractions as is shown in Fig. 2.

### Effect of pre-treatment on molecular weight distribution of soluble macromolecules

**Figure 3:**
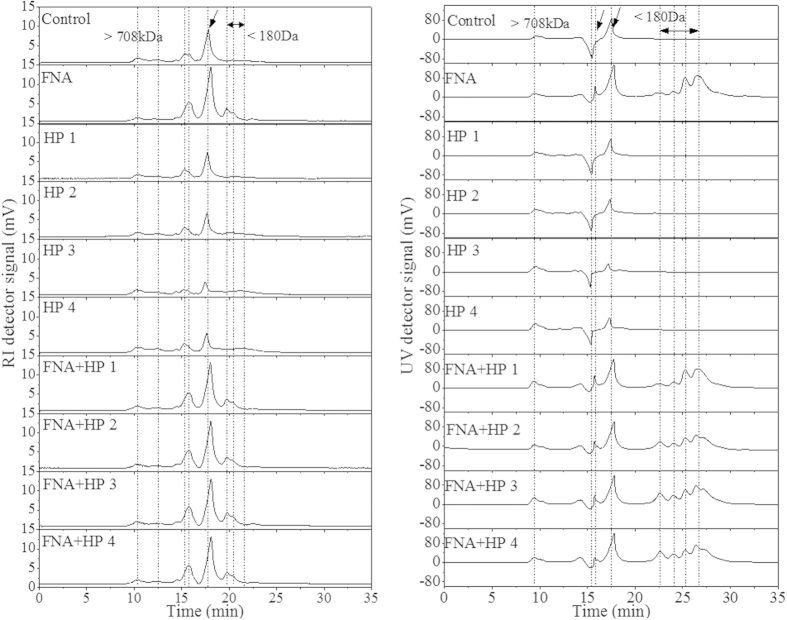
GPC results of macromolecules in soluble phase of WAS with- and without pre-treatment.

Figure 3 shows the molecular weight (MW) distribution of macromolecules in the soluble phase of WAS, with- and without chemical pre-treatment. UV and RI detectors were used to provide MW distribution of UV non-absorbing (carbohydrate) and absorbing (protein and nucleic acids) constituents with the signal intensity used for semi-quantification.

Based on the calibration curve, the peaks emerging no later than t = 10.2 min in the RI spectra represented the macromolecules with MW higher than 708 kDa and the peaks that appeared after 20 min represented the molecules with MW lower than 180 Da. The peaks between 12 min and 20 min arose from the molecules with MW ranging from 708 kDa to 180 Da. In comparison to the sludge without any pre-treatment, the peak at 12.5 min disappeared after FNA- and the combined pre-treatment. The peaks at 15.1 min and at 17.5 min both shifted to lower MW regions after FNA- and the combined pre-treatment. New peaks also emerged in the low-MW region after FNA- and the combined pre-treatment. The peak shifts and new peaks indicate the breakdown of larger molecules into smaller ones. Similar peak shifts were also observed in the UV spectra, with new peaks emerging in the low-MW region with FNA pre-treatment alone and with the combined pre-treatment. In contrast, peak shifts to the higher MW region were observed from both detectors with H_2_O_2_ pre-treatment alone, indicating the likely aggregation of small molecules through anionic functional groups, hydrogen bonding or hydrophobic interaction^21^,^22^. The peaks from both detectors with FNA- and the combined pre-treatment were substantially intensified in comparison to those with H_2_O_2_ pre-treatment alone, suggesting a higher degree of sludge/particulate substance solubilisation with FNA- and the combined pre-treatment than with H_2_O_2_ pre-treatment alone.

### Effect of pre-treatment on molecular structure of soluble macromolecules

**Figure 4:**
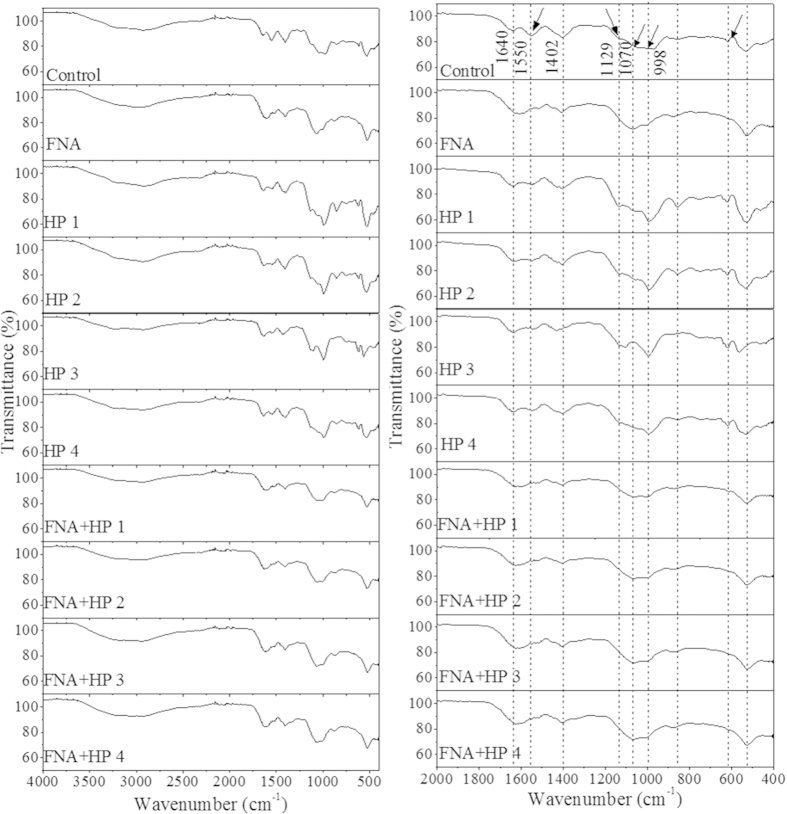
FTIR spectra of macromolecules in soluble phase of WAS with- and without pre-treatment.

Figure 4 shows the FTIR spectra of macromolecule chemical structures in the soluble phase of WAS with- and without chemical pre-treatment. The broad peak near 3300 cm^−1^ corresponds to both υO-H and υN-H stretching. The peak at 1550 cm^−1^, representing δN-H and υ_s_C-N stretching, together with the peak near 3300 cm^−1^ indicates the existence of amide II associated with proteins. The peak at 1550 cm^−1^ was weakened after pre-treatment with H_2_O_2_ alone, and almost disappeared after the FNA- and combined pre-treatment, which is presumably due to the deamination effects of H_2_O_2_, FNA/its derivatives and/or the products of the combination of FNA and H_2_O_2_. The peak at around 1402 cm^−1^ was assigned to υ_s_COO^−^ stretching of carboxylate groups attributed to the presence of uronic and humic acids. H_2_O_2_ pre-treatment alone did not cause obvious changes to this peak, whereas FNA and the combined pre-treatment slightly weakened the peak. The shoulder peak around 1129 cm^−1^ represented the ring vibrations υP = O, υC-O-C, υC-O-P in phosphodiesters and polysaccharides. It was enhanced first as the H_2_O_2_ level was increased to 50 mg/g TS but then decreased as the dosage was further increased. However, after FNA- and the combined pre-treatment, the shoulder peak completely disappeared, implying the oxidation of the ring structures in phosphodiesters and polysaccharides. The same trend was observed for the peak around 998 cm^−1^ representing υ_as_O-P-O stretching associated with nucleic acids. The peak assignment is summarized in Table S1.

## Discussion

All pre-treatments led to an increase in methane potential despite the fact that SCOD after H_2_O_2_ pre-treatment was not consistently higher than that of the control (see Fig. S7). Previous studies have indicated that the SCOD increase is not sufficient to explain the increased methane potential^23^,^24^. Some non-biodegradable fractions have been converted to biodegradable fractions, which accounts for the increased methane potential in comparison with the control. This is supported by the intensified FTIR peaks, which implied the release of inclusions and extracellular substances caused by cell lysis and EPS matrix disruption as the dosage of H_2_O_2_ increased to 50 mg/g TS. The subsequent decline of those peaks at 80 mg H_2_O_2_/g TS could be attributed to the further breakdown of the soluble substances at higher H_2_O_2_ dosages, as demonstrated in the literature that H_2_O_2_ is capable of degrading chitosan and enzymes^25^,^26^. The substantial decline in peak intensity in the MW distribution after H_2_O_2_ pre-treatment alone also provided evidence for H_2_O_2_-induced breakdown of molecules.

The FNA pre-treatment alone (1.54 mg HNO_2_-N/L) increased methane potential substantially by 56%. The extensive SCOD increase after FNA pre-treatment demonstrates the effectiveness of FNA/its derivatives in solubilizing and disintegrating the sludge. The MW distribution analysis of the sludge soluble phase confirmed the significant breakdown of macromolecules into small molecules after FNA pre-treatment in comparison with that of control- and H_2_O_2_ pre-treatment alone, as indicated by the shifts of the peaks and the intensified signals. The chemical structure analysis of the sludge soluble phase revealed that the deamination occurred in the FNA pre-treatment systems. It has been demonstrated that deamination is essential for extensive decomposition of macromolecules^27^ and subsequently impacts on their biodegradability.

Combined pre-treatment resulted in a further increase in SCOD, suggesting that the combinations of FNA and H_2_O_2_ were more effective in sludge/particulates solubilisation. In addition, much higher methane potential (59–83%) was achieved with the combined pre-treatment compared with FNA pre-treatment alone. The chemical structure changes and MW distribution changes with the combined pre-treatment are very similar to those with FNA pre-treatment alone but with stronger intensities, indicating that the addition of a small amount of H_2_O_2_ could significantly reinforce the deaminative and oxidative effects of FNA on organic matters. It has been reported that the reaction between FNA and H_2_O_2_ could produce peroxynitrite followed by decomposition into hydroxyl radical (OH•) and dioxide radical (NO_2_•)^28^. The formation of HO^•^••• NO_2_^•^ at pH 5.5 is able to damage DNA and causes degradation of proteins^19^. This corroborates the results from the modelling showing the conversion of non-biodegradable substances into rapidly biodegradable substances with the pre-treatment, which are likely responsible for the improved methane production.

It has been proposed and demonstrated that both FNA and H_2_O_2_ can be produced as a by-product of wastewater treatment through partial nitritation of the anaerobic digestion liquor with low chemical and energy input^9^,^29^ and a bio-electrochemical system^13^, respectively. In order to assess the potential economic feasibility of the proposed, combined pre-treatment at full-scale, a desktop scaling-up study on a full-scale wastewater treatment plant with a population equivalent (PE) of 400,000 and with an anaerobic sludge digester at a hydraulic retention time (HRT) of 20 days (See Table S2 for details) was employed. As shown in Fig. 1, WAS pre-treatment at the combination of 1.54 mg HNO_2_-N/L and 50 mg H_2_O_2_/g TS achieved the highest methane production at a digestion time of 20 d. Therefore, the increase of methane production at 50 mg H_2_O_2_/g TS (25%), at 1.54 mg HNO_2_-N/L (60%), and at their combination (90%) was selected for the following economic analyses. It has been demonstrated that the performance of the technology on methane production at full-scale can be conservatively estimated by the results from laboratory BMP tests^20^.

The net economic benefits are estimated to be around $-24,000, $357,000 and $427,000 per annum with the respective 25%, 60% and 90% increase in methane production in comparison with the control system (without chemical pre-treatment) (see SI for the detailed calculations). Combined pre-treatment achieves an annual economic outcome that is 13% ($46,000) higher than the FNA pre-treatment alone, and is significantly more profitable in comparison with the H_2_O_2_ pre-treatment alone. Therefore, the combined pre-treatment with FNA and H_2_O_2_ is economically attractive. However, due to the fact that the results are affected by many varied factors such as WAS properties, direct quantitative economic comparison with other available technologies is not feasible at this stage^5^. Also, the effect of the pre-treatment on the dewaterability of the anaerobically digested sludge was not investigated or taken into account in the economic analysis because the large amount of inoculum (inoculum to WAS ratio was 2.0 on a dry VS basis) used in BMP tests would mask these effects. Full-scale tests are required to assess the dewaterability of the anaerobically digested WAS subject to the pre-treatment with the combination of FNA and H_2_O_2_. The costs and benefits presented in this study should be regarded as a reference since this is a proof-of-concept study and the prerequisite for future full-scale study. Also, this study did not aim at the condition optimization (e.g. FNA and H_2_O_2_ concentrations, treatment time). Technology optimization may lead to an even higher methane production, thereby further enhancing the economic benefits of this pre-treatment strategy.

## Methods

### Sludge sources and their characteristics

WAS and the inoculum used for BMP tests were collected respectively from the dissolved air flotation thickener and a mesophilic anaerobic digester of a local biological nutrient removal wastewater treatment plant (WWTP) with sludge retention time (SRT) of 15 days. WAS characteristics were as follows: total solids (TS) 36.1 ± 0.1 g/L, volatile solids (VS) 29.9 ± 0.1 g/L, total chemical oxygen demand (TCOD) 42.3 ± 0.2 g/L, soluble chemical oxygen demand (SCOD) 0.39 ± 0.02 g/L, pH = 6.4 ± 0.0. The inoculum characteristics were as follows: TS 21.2 ± 0.2 g/L, VS 15.3 ± 0.1 g/L, TCOD 24.3 ± 0.1 g/L, SCOD 0.59 ± 0.03 g/L, pH = 7.5 ± 0.0.

### Pre-treatment of WAS with FNA alone, H_2_O_2_ alone, and combined FNA and H_2_O_2_

Batch tests were set up to assess and compare the effect of FNA, H_2_O_2_ and FNA + H_2_O_2_ pre-treatment on the characteristics of WAS. 2.0 L of WAS was evenly distributed into ten batch reactors. Each test lasted for 24 h. For FNA pre-treatment, pH was controlled at 5.5 ± 0.2 via a programmable logic controller using 1.0 M HCl solution. A nitrite stock solution (40 g N/L) was added to a batch reactor to achieve the designated nitrite concentration of 200 mg N/L to give rise to the FNA concentration of 1.54 mg N/L, which was calculated using the formula 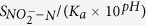 with the K_a_ value determined as a function of temperature T (°C) (22 °C in this study) with the formula 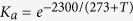
30. The FNA concentration and pre-treatment duration have been demonstrated to be effective in enhancing methane production^11^; therefore, these conditions were employed in this study. For H_2_O_2_ pre-treatment, pH was monitored (between 6.4 and 6.9) but not controlled during the pre-treatment. The H_2_O_2_ stock solutions (30% w/v) was added to four batch reactors in different volumes to achieve the designated H_2_O_2_ concentrations varying between 10 and 80 mg/g TS, as summarized in Table 2. The H_2_O_2_ concentrations used in this study were selected based on an estimated economic analysis. The combined FNA and H_2_O_2_ pre-treatment was carried out under the conditions according to Table 2. The previous study has shown that sludge pre-treatment at pH 5.5 did not have significant effect on the sludge flocs/particulate solubilisation and the chemical structures of the macromolecules in the soluble phase^31^. Therefore, a control reactor without chemical addition or pH control was also set up. All the reactors were well mixed with magnetic stirrers. The control reactor was also stirred at the same intensity as the other reactors to neutralize the influence of stirring on the cell lysis.

### Anaerobic biochemical methane potential (BMP) tests

Methane production from WAS with- and without pre-treatment was assessed using BMP tests (All tests were in triplicate), as described in Jensen, *et al.*^32^. 160 mL serum bottles (100 mL working volume) were used with 80 mL inoculum and 20 mL WAS at an inoculum to substrate ratio of 2:1 (VS basis). WAS and the inoculum were added and well mixed in the bottles. The pH in all BMP bottles was similar in the range of 7.0 – 7.4, which was in the optimum pH range for methanogens^33^. Then the bottles were immediately sealed with butyl rubber stoppers and aluminium crimp-caps after flushing thoroughly with nitrogen gas and stored in a temperature controlled incubator at 37 ± 1 °C. Blanks were set up with the inoculum and MilliQ water at the same ratio as the inoculum to substrate. The tests lasted for over 44 days until the biogas production dropped to insignificant levels. It should be noted that FNA concentration was diluted significantly to around 0.01 mg N/L in the BMP bottles after mixing the inoculum with WAS. The residual amount of FNA can be efficiently removed by denitrification within a few hours. Therefore, it is believed that FNA in BMP bottles would not inhibit the methanogenic activities, which has also been confirmed in our previous study^11^.

### Biochemical methane potential (BMP) modelling

A two-substrate model was used to investigate the effects of pre-treatment on sludge biodegradability by separating the methane potential based on the contribution from rapidly- and slowly biodegradable fractions in WAS^34^ as shown in equation (1):


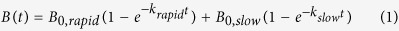


Where B_0,rapid_ is the biochemical methane potential of the rapidly biodegradable substrates (L CH_4_/kg VS added); k_rapid_ is the hydrolysis rate of the rapidly biodegradable substrates (d^−1^); B_0,slow_ is the biochemical methane potential of the slowly biodegradable substrates (L CH_4_/kg VS added); k_slow_ is the hydrolysis rate of the slowly biodegradable substrates (d^−1^). The combined B_0_ was estimated from the sum of the rapid- and slow fractions.

The model was implemented in a modified version of Aquasim 2.1d with sum of squared errors (J_opt_ = RSS) as an objective function^32^. All parameters were simultaneously estimated using the gradient search method in Aquasim 2.1 d. The uncertainty surfaces of k and B_0_, based on a model-validity F-test with 95% confidence limits, were estimated^20^. Confidence intervals (95%) in parameter values were also expressed based on a two-tailed t-test using the estimated standard error in parameter value. The degradation extent (Y) of WAS was determined using equation (2):


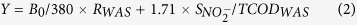


Where B_0_ is the biochemical methane potential (L CH_4_/kg VS added); 380 is the theoretical biochemical methane potential under standard conditions (25 °C, 1 atm) (L CH_4_/kg TCOD)^35^; R_WAS_ is the measured ratio of VS to TCOD in WAS (0.71 in this study); 1.71 is the oxygen equivalent of nitrite (kg O_2_/kg NO_2_^−^–N)^36^,^37^; 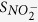 is the measured nitrite concentration in WAS after pre-treatment (g N/L) (0.04 g N/L in this study); TCOD_WAS_ is the COD of WAS after pre-treatment (8.5 g COD/L in this study).

### Analysis

TS, VS, TCOD and SCOD were determined according to the standard methods^38^. The supernatant of WAS samples, centrifuged at 5000 g for 15 minutes, was filtered through disposable Millipore filter units (0.45 μm pore size) for the chemical analyses and structural analyses. The NH_4_^+^-N, NO_2_^−^-N and soluble Kjeldahl nitrogen (SKN) concentrations were analyzed using a Lachat QuikChem8000 Flow Injection Analyzer (Lachat Instrument, Milwaukee, Wisconsin) both prior to- and after pre-treatment and then expressed as a biomass specific value divided by the corresponding VS of WAS measured before the pre-treatment. TS and VS of WAS did not change significantly after the pre-treatment (P > 0.05), VS of WAS measured before the pre-treatment was used in order to normalize the changes of the aforementioned parameters.

MW distribution and chemical structure of the macromolecules in the supernatant phase were analyzed by gel permeation chromatography (GPC) and Fourier transform infrared spectroscopy (FTIR) with the methods described in Zhang, *et al.*^31^.

The biogas (CH_4_ and CO_2_) volume from BMP tests was determined using a manometer and the composition was analyzed using a Shimadzu GC-2014 gas chromatograph equipped with a Valco GC valve and a thermal conductivity detector. Cumulative volumetric gas production was calculated from the pressure increase in the headspace volume (60 mL) and expressed under standard conditions (25 °C, 1 atm). The methane production from WAS was calculated by deducting measured biogas production from the blank and expressed as the volume of methane produced per kilogram of VS added (L CH_4_/kg VS added). Since it has been demonstrated in our previous study that the nitrite concentration used in this study did not have a significant effect on the performance of inoculum, therefore the blank used in this study was considered valid for methane production correction for all tests^11^.

## Additional Information

**How to cite this article**: Zhang, T. *et al.* Combined free nitrous acid and hydrogen peroxide pre-treatment of waste activated sludge enhances methane production via organic molecule breakdown. *Sci. Rep.* **5**, 16631; doi: 10.1038/srep16631 (2015).

## Supplementary Material

Supplementary Information

## Figures and Tables

**Table 1:** Estimated k_rapid_, B_0,rapid_, Y_rapid_ and k_slow_, B_0,slow_, Y_slow_, B_0,total_ at different pre-treatment conditions using two-substrate model (with 95% confidence intervals).

Pre-treatment	k_rapid_ (d^−1^)	B_0,rapid_ (L CH_4_/kgVS added)	Y_rapid_	k_slow_ (d^−1^)	B_0,slow_ (L CH_4_/kgVS added)	Y_slow_	B_0,total_ (L CH_4_/kgVS added)
Control	0.97 ± 0.09	70 ± 3	0.13 ± 0.01	0.06 ± 0.01	94 ± 2	0.17 ± 0.01	164 ± 3
FNA	0.63 ± 0.03	136 ± 4	0.25 ± 0.01	0.05 ± 0.01	124 ± 3	0.23 ± 0.01	260 ± 5
HP1	1.10 ± 0.11	77 ± 3	0.14 ± 0.01	0.06 ± 0.01	108 ± 2	0.20 ± 0.01	185 ± 4
HP2	1.13 ± 0.10	99 ± 3	0.18 ± 0.01	0.06 ± 0.01	102 ± 2	0.19 ± 0.01	201 ± 4
HP3	1.05 ± 0.08	95 ± 3	0.18 ± 0.01	0.06 ± 0.01	104 ± 2	0.20 ± 0.01	199 ± 3
HP4	0.90 ± 0.09	85 ± 4	0.16 ± 0.01	0.06 ± 0.01	108 ± 3	0.20 ± 0.01	193 ± 5
FNA + HP1	0.63 ± 0.03	141 ± 4	0.26 ± 0.01	0.05 ± 0.01	124 ± 2	0.23 ± 0.01	265 ± 4
FNA + HP2	0.53 ± 0.03	162 ± 5	0.30 ± 0.01	0.04 ± 0.01	117 ± 4	0.22 ± 0.01	279 ± 6
FNA + HP3	0.46 ± 0.02	185 ± 5	0.35 ± 0.01	0.04 ± 0.01	125 ± 4	0.24 ± 0.01	310 ± 6
FNA + HP4	0.48 ± 0.02	174 ± 6	0.33 ± 0.01	0.04 ± 0.01	122 ± 4	0.23 ± 0.01	296 ± 7

**Table 2:** Pre-treatment conditions applied in this study.

Reactor No.	Pre-treatment	FNA (mg N/L)	H_2_O_2_ concentration (mg/g TS)	NO_2_^−^-N (mg N/L)	pH
1	Control	0	0	0	6.4–6.9
FNA pre-treatment					
2	FNA	1.54	0	200	5.5 ± 0.2
H_2_O_2_ pre-treatment					
3	HP1	0	10	0	6.4–6.9
4	HP2	0	30	0	6.4–6.9
5	HP3	0	50	0	6.4–6.9
6	HP4	0	80	0	6.4–6.9
Combined FNA and H_2_O_2_ pre-treatment					
7	FNA + HP1	1.54	10	200	5.5 ± 0.2
8	FNA + HP2	1.54	30	200	5.5 ± 0.2
9	FNA + HP3	1.54	50	200	5.5 ± 0.2
10	FNA + HP4	1.54	80	200	5.5 ± 0.2
