# Supplementary material for: Combined free nitrous acid and hydrogen peroxide pre-treatment of waste activated sludge enhances methane production via organic molecule breakdown
Source: Sci Rep. 2015 Nov 13;5:16631. doi: 10.1038/srep16631 (PMC4643222; doi:10.1038/srep16631)
Supplement: Supplementary Information [file srep16631-s1.pdf]

Combined free nitrous acid and hydrogen peroxide pre-treatment of waste activated sludge enhances methane production via organic molecule breakdown

SUPPLEMENTARY INFORMATION

Tingting Zhang[1], Qilin Wang[1,*], Liu Ye[1,2], Damien Batstone[1] and Zhiguo Yuan[1,*]

[1]Advanced Water Management Centre (AWMC), The University of Queensland, QLD 4072, Australia
[2]School of Chemical Engineering, The University of Queensland, QLD 4072, Australia

[*] Corresponding Authors
(Q.W.) Phone: +61 7 33463229; fax: +61 7 33654726; e-mail: q.wang@awmc.uq.edu.au;
(Z.Y.) Phone: +61 7 33654374; fax: +61 7 3365 4726; e-mail: zhiguo@awmc.uq.edu.au

Confidence region (95%) of the estimated parameters from sludge with and without pre-treatment

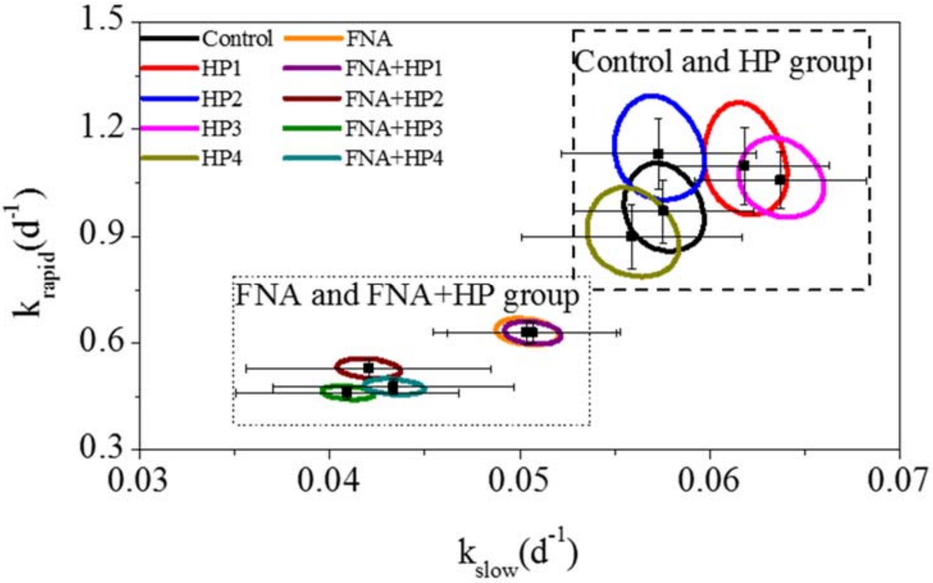

Figure S1 Confidence regions (95%) of the estimated parameters from the sludge with and without pre-treatment: k_{slow} vs. k_{rapid}.

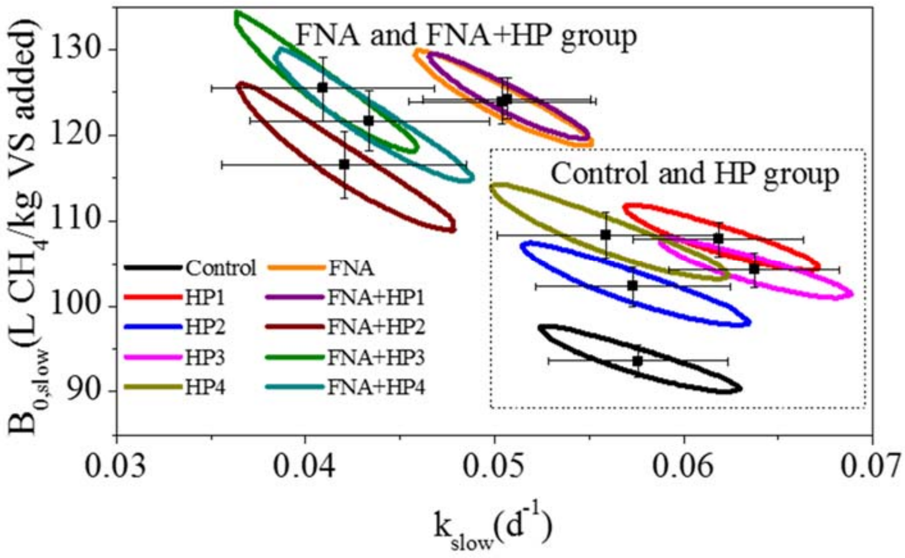

Figure S2 Confidence regions (95%) of the estimated parameters from the sludge with and without pre-treatment: k_{slow} vs. $B_{0,slow}$.

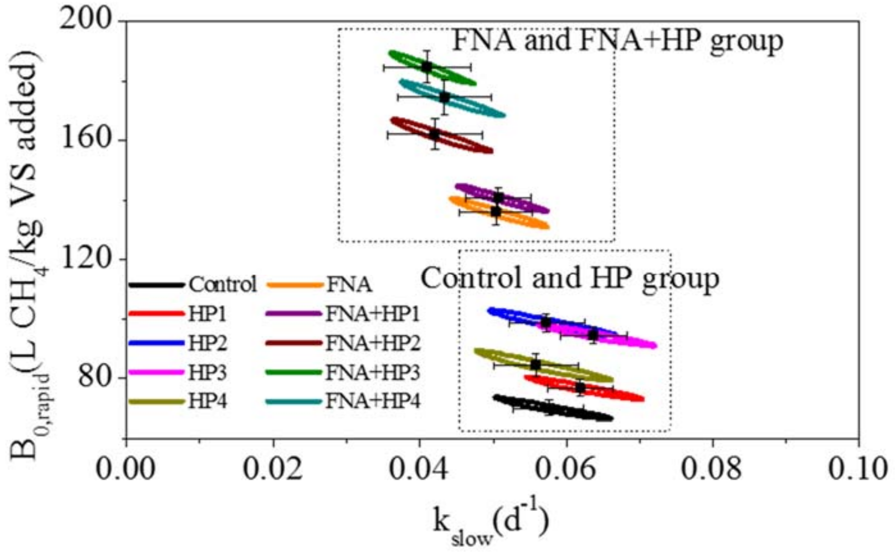

Figure S3 Confidence regions (95%) of the estimated parameters from the sludge with and without pre-treatment: k_{slow} vs. $B_{0,rapid}$.

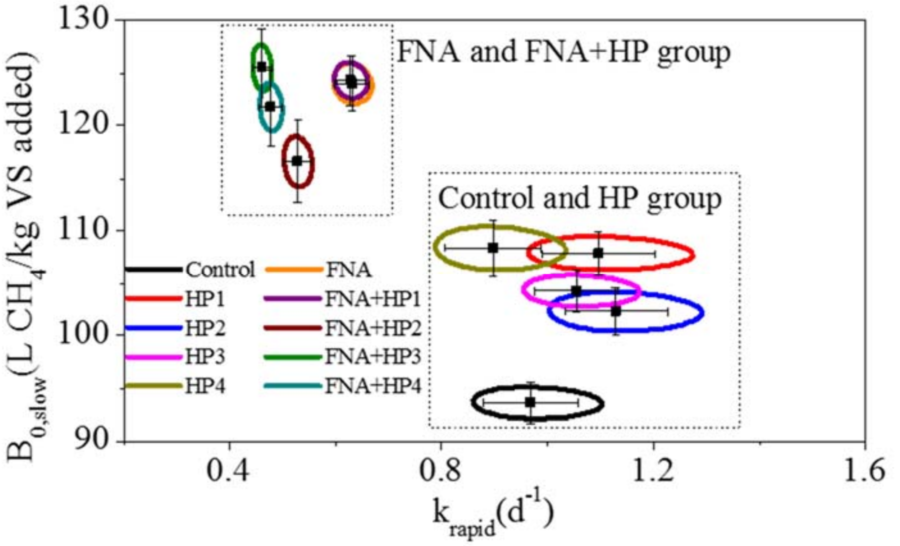

Figure S4 Confidence regions (95%) of the estimated parameters from the sludge with and without pre-treatment: k_{rapid} vs. $B_{0,slow}$.

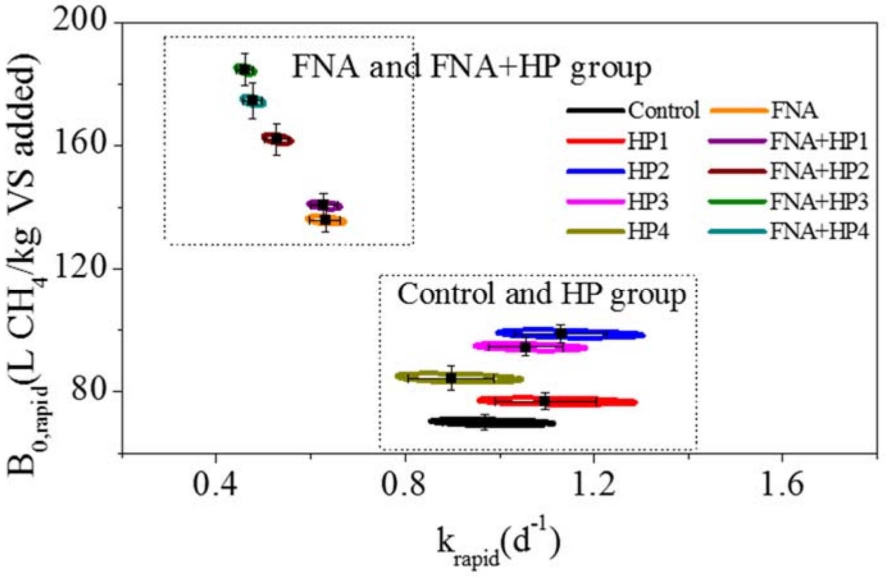

Figure S5 Confidence regions (95%) of the estimated parameters from the sludge with and without pre-treatment: k_{rapid} vs. $B_{0,rapid}$.

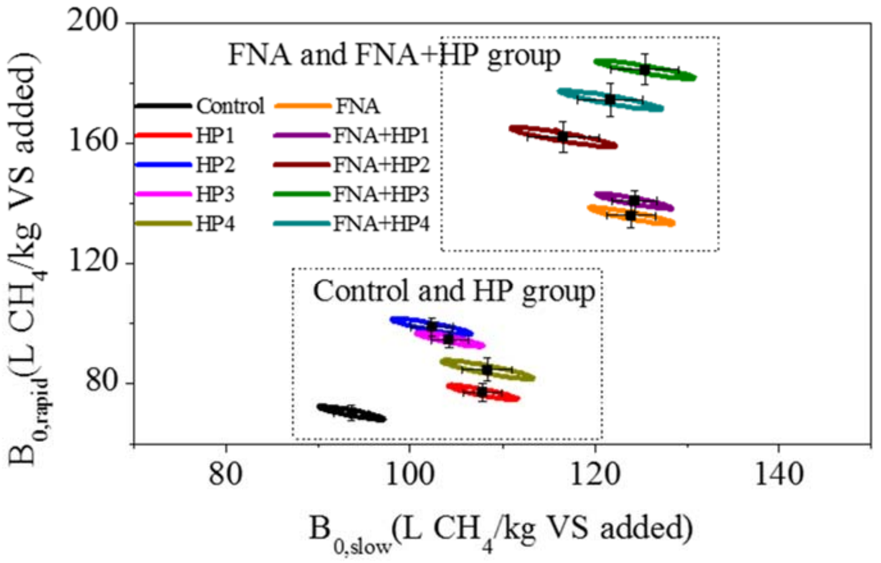

Figure S6 Confidence regions (95%) of the estimated parameters from the sludge with and without pre-treatment: $B_{0,slow}$ vs. $B_{0,rapid}$.

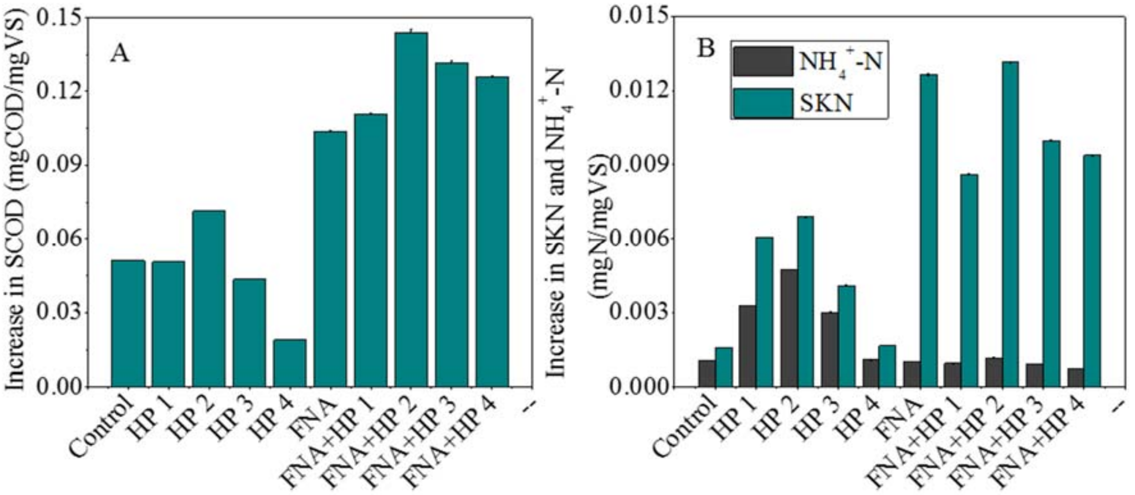

Figure S7 Biomass specific increase of (A) SCOD, (B) NH_4^+-N and SKN after 24 h pre-treatment of WAS. Error bars show standard errors resulting from triplicate tests. See Table 1 for the pre-treatment conditions shown in abscissa. The SCOD of the WAS in the control with only stirring but without any chemical pre-treatment increased by around 0.05 mg COD/mg VS. In contrast, the SCOD of WAS pre-treated with FNA alone and the combinations increased by 0.10-0.14 mg COD/mg VS, with the highest solubilisation achieved in the combined FNA and H_2O_2 pre-treatment (1.54 mg HNO_2-N/L and 30 mg/g TS). Further increasing the H_2O_2 level did not lead to concomitant SCOD increases. In the case of H_2O_2 pre-treatment alone, SCOD increased slightly by 0.07 mg COD/mg VS at the dosage of 30 mg H_2O_2/g TS, but then substantially decreased to 0.01 mg COD/mg VS at the dosage of 80 mg H_2O_2/g TS. The SKN results are consistent with those of SCOD. The decreases in SCOD and SKN at increased H_2O_2 doses are likely due to oxidation of released organics from cell lysis and EPS matrix disruption [1,2]. However, the NH_4^+ results showed a different trend in comparison with those of SCOD and SKN. The NH_4^+ concentration in the WAS treated with H_2O_2 alone increased substantially from 0.0010 mg NH_4^+/mg VS in the control to 0.0047 mg NH_4^+/mg VS at the dosage of 30 mg H_2O_2/g TS and thereafter dropped to that of control. This implies that H_2O_2 was involved in the breakdown of extracellular organics into inorganic fractions at low levels, which accounts for the increase of NH_4^+ [3]. The higher levels of H_2O_2 likely inhibited hydrolytic enzymes, resulting in decreased biological release of NH_4^+ [4,5]. No significant increase was observed with FNA pre-treatment alone and with combined pre-treatment, which is potentially attributed to the inhibitory/toxic effects of FNA and/or the by-products of the reactions between FNA and H_2O_2 on sludge hydrolytic enzymes e.g. protease and/or enzymes responsible for acidogenesis [4-6].

Table S1 FTIR spectra peak assignment [7]

Frequencies (cm^{-1})	Peak assignment
3300	υO-H and υN-H associated with alcohols and amines
1640	$υ_s$C=O stretching (amide I) and υC=N stretching associated with proteins
1550	δN-H and $υ_s$C-N stretching in amide II associated with proteins
1402	$υ_s COO^-$ stretching associated with amino acids
1129	υO-H deformation, υC-O stretching, ring vibration of polysaccharides C-O-C and C-O-P
1070	υC-OH of phosphorylated proteins and polysaccharides
998	$υ_{as}$ O-P-O stretching associated with nucleic acids
900~600	Ring vibrations associated with υC-C and υC-OH from aromatic amino acids and nucleotides

Economic analyses of combined FNA and H_2O_2 pre-treatment for enhancing methane production

A desktop scaling-up study on a full-scale wastewater treatment plant (WWTP) with a population equivalent (PE) of 400,000 and with an anaerobic digester at a hydraulic retention time (HRT) of 20 d was conducted to evaluate the potential economic and environmental benefits of the combined FNA and heat pre-treatment strategy. A system with an annual methane production of approximately 286,000 kg CH_4 was used as a control. The systems with H_2O_2 pre-treatment alone, FNA pre-treatment alone and combined FNA and H_2O_2 pre-treatment were designed to obtain an 25%, 60% and 90% increase in methane production (i.e. 357,500, 457,600 and 543,400 kg CH_4 per annum). The methane produced was considered to be combusted in a cogeneration plant in order to produce both power and heat [8]. The costs/benefits associated with H_2O_2, FNA, and combined FNA and H_2O_2 pre-treatment were estimated and compared, as summarized in Table S2.

Table S2 - Economic and environmental analyses of the H_2O_2, FNA and combined FNA and H_2O_2 pre-treatment for enhancing methane production

**General parameter**	**Values**
Size of the WWTP (Population equivalent - PE)	400,000
Decay coefficient of the heterotrophic biomass (d^{-1})	0.2[a]
Decay coefficient of the nitrifying biomass (d^{-1})	0.1[a]
Yield coefficient of the heterotrophic biomass (g COD/g COD)	0.625[a]
Yield coefficient of the nitrifying biomass (g COD/g N)	0.24[a]
Fraction of inert COD generated in biomass decay (g COD/g COD)	0.2[a]
Mixed liquor suspended solid concentration in the bioreactor (mg/L)	4,000
Mixed liquor volatile suspended solid concentration in the bioreactor (mg/L)	3,200
Sludge retention time (SRT) in the bioreactor of the WWTP (d)	15
Solids content in thickened WAS	5%
Solids content in dewatered WAS	15%
Temperature of WAS (°C)	22
Operating temperature of the anaerobic digester (°C)	37
Hydraulic retention time (HRT) in the anaerobic digester (d)	20
Price of HCl (32%) ($/tonne)	150[b]
Price of $NaHCO_3$ ($/tonne)	145[b]
Price of H_2O_2 (50%) ($/tonne)	450[b]
Mixing energy of the reactor (kwh/(m^3·d))	0.12
Power requirement for oxygen supply (kwh/kgO_2)	0.66[a]
Methane calorific value (kwh/kgCH_4)	16
Power price ($/kwh)	0.15
Conversion efficiency of methane to heat	50%[c]
Conversion efficiency of methane to power	40%[c]
Cost of WAS transport and disposal ($/wet tonne)	50
Period over which capital costs are annualised (i.e. Lifetime) (year)	20
Interest applied for initial capital expenditure	8.5%

**Control system**	Methane production (kg CH_4/y)	216,000
	WAS fed to the anaerobic digester (kg VS/y)	2,420,300
	WAS removal in the anaerobic digester (on a dry VS basis)	25%
**System with H_2O_2 pre-treatment**	Methane production (kgCH_4/y)	270,000
	WAS fed to the anaerobic digester (kg VS/y)	2,420,300
	WAS removal in the anaerobic digester (on a dry VS basis)	32%
	WAS treatment time by H_2O_2 (d)	1
	H_2O_2 concentration in the pre-treatment reactor (g/kg TS)	50
	Capital cost of the H_2O_2 pre-treatment reactor (including major equipments such as pumps) ($)[b]	140,000[e]
	Annualised cost of H_2O_2 pre-treatment reactor ($/y)	15,000
	Annualised power consumption for mixing in H_2O_2 pre-treatment reactor (kwh/y)	7,400
	Annualised mixing cost of H_2O_2 pre-treatment reactor ($/y)	1,100
	Annual cost of H_2O_2 ($/y)	136,000
	Storage time of H_2O_2 (d)	30
	Annualised cost of H_2O_2 storage reactor ($/y)	1,750
	Annualised power consumption for the addition (to the H_2O_2 pre-treatment reactor) of the H_2O_2-treated WAS (kwh/y)	4,800
	Annualised power cost for the addition (to the HP pre-treatment reactor) of the H_2O_2-treated WAS ($/y)	580
	Annual extra heat production from methane conversion (compared to the control system) (kwh/y)	460,000
	Annual extra power production from methane conversion (compared to the control system) (kwh/y)	367,000
	Annual cost associated with WAS pre-treatment ($/y)	154,000
	Annual reduced WAS transport and disposal cost (compared to the control system) ($/y)	54,000
	Annual extra obtained benefit (compared to the control system) due to the extra heat and power generation ($/y)	124,000
	**Annual saving (compared to the control system) ($/y)**	**-24,000**
**System with FNA pre-treatment**	Methane production (kgCH_4/y)	345,000
	WAS fed to the anaerobic digester (kg VS/y)	2,420,300
	WAS removal in the anaerobic digester (on a dry VS basis)	41%
	Biodegradable COD (bCOD) concentration in the anaerobic digestion liquor (mg/L)	300
	HCO_3^-/ NH_4^+-N in the anaerobic digestion liquor (mol/mol)	1
	SRT in the FNA production reactor (d)	11[d]
	HRT in the FNA production reactor (d)	1.33[d]
	Conversion efficiency of NH_4^+-N to NO_2^--N	93%[d]
	Conversion efficiency of NH_4^+-N to NO_3^--N	3%[d]
	WAS treatment time by FNA (d)	1

pH used in the FNA pre-treatment reactor	5.5
Concentration of NO_2^- in the FNA pre-treatment reactor (mg N/L)	200
Temperature in the FNA pre-treatment reactor (°C)	22
Concentration of FNA in the FNA pre-treatment reactor (mg HNO_2-N/L)	1.42
Capital cost of FNA production reactor (including major equipments such as pumps and air compressor) ($)[b]	36,000[e]
Annualised cost of FNA production reactor ($/y)	3,800
Annualised power consumption for mixing in the FNA production reactor (kwh/y)	1,000
Annualised mixing cost of FNA production reactor ($/y)	150
Annualised power consumption for the oxidation of NH_4^+-N and bCOD (kwh/y)	37,000
Annualised power cost for the oxidation of NH_4^+-N and bCOD ($/y)	5,500
Annual cost of $NaHCO_3$ ($/y)	11,500
Storage time of $NaHCO_3$ (d)	10
Capital cost of $NaHCO_3$ storage reactor (including major equipments such as pumps) ($)	35,000[e]
Annualised cost of $NaHCO_3$ storage reactor ($/y)	3,700
Annual cost of HCl ($/y)	12,000
Storage time of HCl (d)	30
Annualised cost of HCl storage reactor ($/y)	950
Capital cost of FNA pre-treatment reactor (including major equipments such as pumps) ($)	152,000[e]
Annualised cost of FNA pre-treatment reactor (including major equipments such as pumps) ($/y)	16,000
Annualised power consumption for mixing in FNA pre-treatment reactor (kwh/y)	8,000
Annualised mixing cost in the FNA pre-treatment reactor ($/y)	1,200
Annualised power consumption for the addition (to the FNA pre-treatment reactor) of the FNA-treated WAS (kwh/y)	4,800
Annualised power cost for the addition (to the FNA pre-treatment reactor) of the FNA-treated WAS ($/y)	720
Annual extra heat production from methane conversion (compared to the control system) (kwh/y)	1,050,000
Annual extra power production from methane conversion (compared to the control system) (kwh/y)	840,000
Annual cost associated with WAS pre-treatment ($/y)	55,500
Annual reduced WAS transport and disposal cost (compared to the control system) ($/y)	129,000
Annual extra obtained benefit (compared to the control system)	283,500

	due to the extra heat and power generation($/y)	
	**Annual saving (compared to the control system) ($/y)**	**357,000**
**System with $FNA+H_2O_2$ pre-treatment**	Methane production (kgCH_4/y)	410,000
	WAS fed to the anaerobic digester (kg VS/y)	2,420,300
	WAS removal in the anaerobic digester (on a dry VS basis)	48%
	Biodegradable COD (bCOD) concentration in the anaerobic digestion liquor (mg/L)	300
	HCO_3^-/ NH_4^+-N in the anaerobic digestion liquor (mol/mol)	1
	SRT in the FNA production reactor (d)	11[d]
	Hydraulic retention time (HRT) in the FNA production reactor (d)	1.33[d]
	Conversion efficiency of NH_4^+-N to NO_2^--N	93%[d]
	Conversion efficiency of NH_4^+-N to NO_3^--N	3%[d]
	WAS treatment time by FNA (d)	1
	pH used in the pre-treatment reactor	5.5
	Concentration of NO_2^- in the pre-treatment reactor (mg N/L)	200
	Concentration of FNA in the pre-treatment reactor (mg HNO_2-N/L)	1.42
	Capital cost of FNA production reactor (including major equipments such as pumps and air compressor) ($)[b]	36,000[e]
	Annualised cost of FNA production reactor ($/y)	3,800
	Annualised power consumption for mixing in FNA production reactor (kwh/y)	1,000
	Annualised mixing cost of FNA production reactor ($/y)	150
	Annualised power consumption for the oxidation of NH_4^+-N and bCOD (kwh/y)	37,000
	Annualised power cost for the oxidation of NH_4^+-N and bCOD ($/y)	5,500
	Annual cost of $NaHCO_3$ ($/y)	11,500
	Storage time of $NaHCO_3$ (d)	10
	Capital cost of $NaHCO_3$ storage reactor (including major equipments such as pumps) ($)	35,000[e]
	Annualised cost of $NaHCO_3$ storage reactor ($/y)	3,700
	Annual cost of HCl ($/y)	12,000
	Storage time of HCl (d)	30
	Annualised cost of HCl storage reactor ($/y)	950
	Annual cost of H_2O_2 ($/y)	136,000
	Storage time of H_2O_2 (d)	30
	Annualised cost of H_2O_2 storage reactor ($/y)	1,750
	Capital cost of pre-treatment reactor (including major equipments such as pumps) ($)	152,000[e]
	Annualised cost of pre-treatment reactor (including major equipments such as pumps) ($/y)	16,000

Annualised power consumption for mixing in the pre-treatment reactor (kwh/y)	8,000
Annualised mixing cost in the pre-treatment reactor ($/y)	1,200
Annualised power consumption for the addition (to the pre-treatment reactor) of the pre-treated WAS (kwh/y)	4,800
Annualised power cost for the addition (to the pre-treatment reactor) of the pre-treated WAS ($/y)	580
Annual extra heat production from methane conversion (compared to the control system) (kwh/y)	1,520,000
Annual extra power production from methane conversion (compared to the control system) (kwh/y)	1,210,000
Annual cost associated with WAS pre-treatment ($/y)	193,000
Annual reduced WAS transport and disposal cost (compared to the control system) ($/y)	186,000
Annual extra obtained benefit (compared to the control system) due to the extra heat and power generation($/y)	410,000
**Annual saving (compared to the control system) ($/y)**	**403,000**
**Annual saving (compared to the FNA pre-treatment system) ($/y)**	**46,000**
**Annual saving (compared to H_2O_2 pre-treatment system) ($/y)**	**427,000**

[a] Refer to Metcalf and Eddy.[9]

[b] http://www.alibaba.com/

[c] Refer to Carballa, et al. [8].

[d]Refer to Law, et al. [10].

[e]The capital cost of the bioreactor was estimated using the following equation.[11] $493601\times(V/1000)^{0.7202}$, where V=volume of the reactor.

References

1 Christensen, B. E., Myhr, M. H. & Smidsrod, O. Degradation of double-stranded xanthan by hydrogen peroxide in the presence of ferrous ions: Comparison to acid hydrolysis. *Carbohyd Res* **280**, 85-99, (1996).

2 Wingender, J., Neu, T. R. & Flemming, H.-C. *Microbial extracellular polymeric substances: characterization, structure and function*. 231-251, (Springer, 1999).

3 Qin, C. Q., Du, Y. M. & Xiao, L. Effect of hydrogen peroxide treatment on the molecular weight and structure of chitosan. *Polym Degrad Stabil* **76**, 211-218, (2002).

4 Denu, J. M. & Tanner, K. G. Specific and reversible inactivation of protein tyrosine phosphatases by hydrogen peroxide: evidence for a sulfenic acid intermediate and implications for redox regulation. *Biochemistry* **37**, 5633-5642, (1998).

5 Hodgson, E. K. & Fridovich, I. The interaction of bovine erythrocyte superoxide dismutase with hydrogen peroxide: inactivation of the enzyme. *Biochemistry* **14**, 5294-5299, (1975).

6 Pijuan, M., Wang, Q. L., Ye, L. & Yuan, Z. G. Improving secondary sludge biodegradability

using free nitrous acid treatment. *Bioresour technol* **116**, 92-98, (2012).

7 Zhang, T., Wang, Q., Khan, J. & Yuan, Z. Free nitrous acid breaks down extracellular polymeric substances in waste activated sludge. *Roy Soc Ch Adv* **5**, 43312-43318, (2015).

8 Carballa, M., Duran, C. & Hospido, A. Should we pretreat solid waste prior to anaerobic digestion? An assessment of its environmental cost. *Environ Sci Technol* **45**, 10306-10314, (2011).

9 Tchobanoglous, G., Stensel, H. D., Tsuchihashi, R. & Burton, F. L. *Wastewater Engineering: Treatment and Resource Recovery, 5th Ed.* (Metcalf & Eddy I AECOM, McGraw-Hill Book Company, 2014).

10 Law, Y. Y., Ye, L., Wang, Q.L., Hu, S.H., Pijuan, M., Yuan, Z.G.. Producing free nitrous acid - A green and renewable biocidal agent - From anaerobic digester liquor. *Chem Eng J* **259**, 62-69, (2015).

11 Hartley, K. J. The Cost of Australian BNR Plants. *Water -Melbourne Then Artarmon* **25**, 20-22, (1998).
